# Physical Activity Is Prospectively Associated With Adolescent Nonalcoholic Fatty Liver Disease

**DOI:** 10.1097/MPG.0000000000000904

**Published:** 2015-08-04

**Authors:** Emma L. Anderson, Abigail Fraser, Laura D. Howe, Mark P. Callaway, Naveed Sattar, Chris Day, Kate Tilling, Debbie A. Lawlor

**Affiliations:** ∗ MRC Integrative Epidemiology; † School of Social and Community Medicine, University of Bristol; ‡ University Hospitals Bristol NHS Foundation Trust, Bristol; § Institute of Cardiovascular and Medical Sciences, BHF Glasgow Cardiovascular Research Centre, Faculty of Medicine, University of Glasgow, Glasgow; || Institute of Cellular Medicine, Faculty of Medical Sciences, Newcastle University, Newcastle upon Tyne, UK.

**Keywords:** Avon Longitudinal Study of Parents and Children, childhood, exercise, fatty liver, physical activity

## Abstract

**Objectives:**

The aim of the present study was to assess whether objectively measured physical activity at mean ages 12 and 14 years are prospectively associated with ultrasound scan liver fat and stiffness (alanine aminotransferase, aspartate aminotransferase [AST], and γ-glutamyl transferase [GGT]) assessed at mean age 17.8 years.

**Methods:**

Participants were from the Avon Longitudinal Study of Parents and Children. Total physical activity (counts per minute) and minutes of moderate to vigorous physical activity (MVPA) were measured using ActiGraph accelerometers at mean ages 12 and 14 years.

**Results:**

Greater total physical activity and MVPA at ages 12 and 14 years were associated with lower odds of liver fat and lower GGT levels at mean age 17.8 years, such as per 15-minute increase in daily MVPA at age 12 years, the confounder adjusted odds ratio of liver fat was 0.47 (95% confidence interval [CI] 0.27–0.84). Associations attenuated after additional adjustment for fat mass as a potential confounder (eg, per 15-minute increase in daily MVPA at age 12 years, the odds ratio of liver fat attenuated to 0.65 [95% CI 0.35–1.21]) or a potential mediator (eg, per 15-minute increase in daily MVPA at age 12 years the odds ratio of liver fat attenuated to 0.59 [95% CI 0.32–1.09]). Results did not further attenuate after additional adjustment for insulin resistance. There was some evidence that greater total physical activity and MVPA at age 12 years were associated with the higher AST levels.

**Conclusions:**

Adolescents who were more active in childhood have lower odds of fatty liver and lower GGT levels. These findings are likely to be, at least in part, explained by adiposity.

**What Is Known**

- Cross-sectional evidence suggests physical activity is inversely related to nonalcoholic fatty liver disease in children/adolescents.
- Prospective associations have not been examined. Thus, we are unable to establish whether lower physical activity causes nonalcoholic fatty liver disease or nonalcoholic fatty liver disease causes lower physical activity.

**What Is New**

- We provide evidence that adolescents who are more active in late childhood have lower risk of ultrasound scan fatty liver and lower γ-glutamyl transferase levels.
- These findings are likely to be, in part, explained by adiposity.
- If replicated, our findings highlight the importance of maintaining healthy levels of physical activity throughout childhood for preventing nonalcoholic fatty liver disease.

Greater adiposity is an important risk factor for nonalcoholic fatty liver disease (NAFLD) (1). Consequently, lifestyle modifications, including increasing physical activity levels with the aim of reducing adiposity and limiting fatty liver infiltration and necroinflammation, are recommended for both adult and child patients with NAFLD (2,3). Physical activity could influence NAFLD risk in several ways. Physical inactivity is associated with greater total adiposity (4), which may increase the risk of fat infiltration into hepatocytes by increasing free fatty acid (FFA) influx from adipose tissue to the liver (5). Physical activity has been shown to improve insulin sensitivity through decreasing fasting insulin and increasing lean mass (6). In an insulin-resistant state, the ability of insulin to suppress adipose tissue lipolysis is impaired, leading to an increased efflux of FFA from adipose tissue and an increased delivery of FFA to the liver (7). Physical activity may also reduce levels of inflammatory markers that are involved in the progression of steatosis to nonalcoholic steatohepatitis (8,9).

The existing evidence for an inverse association between physical activity and NAFLD comes from cross-sectional studies using questionnaires to assess physical activity (10–18), thus potentially underestimating associations in comparison with objectively measured physical activity (19). Furthermore, cross-sectional studies cannot rule out reverse causation (ie, greater adiposity and associated NAFLD causing people to be less active rather). Two recent studies support this possibility (20,21). We are unaware of any prospective studies assessing associations of physical activity with NAFLD in children and/or adolescents. We aimed to assess whether objectively measured physical activity at mean ages 12 and 14 years are associated with markers of NAFLD assessed at mean age 17.8 years, and to determine whether any observed associations are mediated through fat mass and insulin.

## METHODS

### Study Population

**FIGURE 1:**
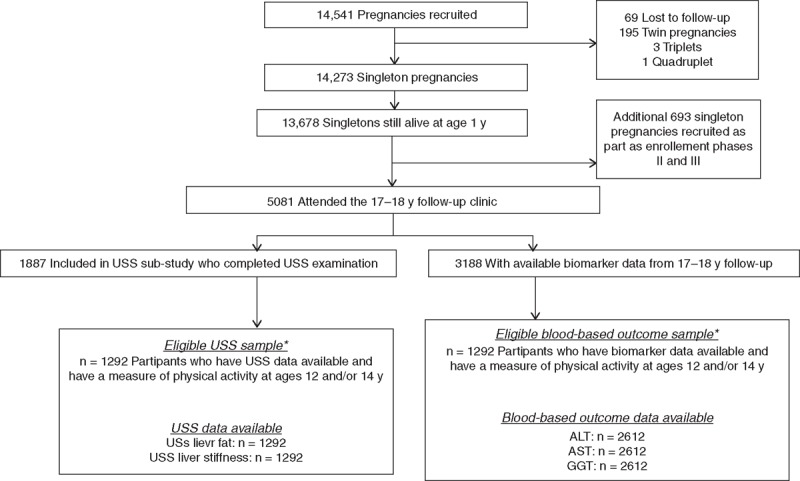
Participant flow through the study. ^∗^Participants were excluded if they had no measure of physical activity measure at 12 or 14 years, or they had harmful alcohol consumption. ALT = alanine aminotransferase; AST = aspartate aminotransferase; GGT = γ-glutamyl transferase; USS = ultrasound scan.

Avon Longitudinal Study of Parents and Children (ALSPAC) is a prospective birth cohort from southwest England (full details in online supplement) (22,23). The study website contains details of all available data through a fully searchable data dictionary (*www.bris.ac.uk/alspac/researchers/data-access/data-dictionary*). Ethical approval for the study was obtained from the ALSPAC Ethics and Law Committee and the local research ethics committees. Briefly, ALSPAC recruited a cohort of 14,541 pregnancies with expected delivery dates between April 1, 1991, and December 31, 1992. A total of 13,678 singleton live-born infants resulted from these pregnancies. The cohort has been followed-up since birth, including repeat clinical assessment from age 7 years to capture information on a range of characteristics such as demographics, health-related behaviors, psychological and social wellbeing, and health factors. A total of 5081 participants attended the 17- to 18-year follow-up assessment at mean age 17.8 years, and of these, N = 3188 (62.7%) had data available for blood-based indicators of liver function including alanine aminotransferase (ALT), aspartate aminotransferase (AST), and/or GGT. A liver ultrasound scan substudy was undertaken on a randomly selected subgroup of participants attending the 17- to 18-year follow-up (N = 1887, 37%, Fig. 1) (24).

None of the participants had a known history of jaundice or hepatitis, were taking medications or receiving treatment that would indicate they had hepatic disease, or were taking medication known to influence liver function. Participants’ alcohol consumptions were assessed at mean ages 16.7 and 17.8 years using the Alcohol Use Disorders Identification Test (AUDIT), which has been used to validate the assessment of alcohol consumption in adolescents (25). Participants answered 10 questions about their alcohol consumption, and from their responses, a score between 0 and 20 was derived. A score >16 is classified as harmful alcohol consumption. Ten participants who completed the ultrasound scan examination and 25 participants with blood-based liver data who were classified as harmful drinkers at both ages were excluded from this study.

### Outcome Assessment

#### Ultrasound Scan Assessment of Liver Fat and Stiffness

Details of ultrasound scan assessment in ALSPAC have been published and are in the online supplement (24). Briefly, upper abdominal ultrasound scan was completed by 1 of 4 trained sonographers using an Acuson S2000 ultrasound scan system (Siemens, Erlangen, Germany) to assess echogenicity and liver stiffness (our main indicator of liver fibrosis). Echogenicity was assessed during deep inspiration and recorded as present, absent, or uncertain according to established protocols (26,27). Thus, the binary ultrasound scan liver fat variable in this study is coded as present or absent. Acoustic radiation force impulse (ARFI) imaging of the right lobe of the liver was used to measure liver stiffness using standard protocols (28,29). Levels of agreement in identifying echogenicity between the 4 sonographers was high, both immediately after training and at 6-month intervals during data collection (absolute agreement of 98% or greater).

#### Assessment of Blood-Based Liver Outcomes

Participants were instructed to fast overnight or for a minimum of 6 hours. Fasting blood samples were immediately spun and frozen at −80°C. Measurements were assayed within 3 to 9 months after samples were taken, with no previous freeze-thaw cycles. ALT, GGT, and AST were measured by automated analyzers with enzymatic methods. All of the inter coefficients and intracoefficients of variation for these blood-based assays were <5%.

### Assessment of Physical Activity

Detailed methods of physical activity assessment have been described (30) and are in the online supplement. Briefly, physical activity was objectively assessed at mean ages 12 and 14 years with a uniaxial ActiGraph accelerometer (AM7164 2.2; ActiGraph LLC, Fort Walton Beach, FL; *http://www.theactigraph.com*) for 7 days. The participants recorded the times at which the ActiGraph was worn and any times that they swam or cycled each day. Total physical activity (average counts per minute) during the valid measurement period and average time spent in moderate to vigorous physical activity (MVPA) in minutes per valid day were derived. The cutoff point used to define MVPA was a CPM >3600 (31). Counts are a result of summing postfiltered accelerometer values (raw data at 30 Hz) into epoch “chunks.” The value of the counts will vary based on the frequency and intensity of the raw acceleration. The filtering process by which counts are produced is proprietary to ActiGraph.

### Covariables

The assessment of all of the covariables is described in detail in the online supplement. The following were considered potential confounders: sex, maternal age at delivery, parity, maternal education, head of household, social class, maternal body mass index (BMI), ethnicity, energy intake at age 11 years, pubertal status at age 11 years, age at physical activity assessments and liver outcome assessment, and length of time accelerometer was worn (in minutes). Given that adiposity is known to be causally related to physical activity (20), and could possibly be causally related to NAFLD, we considered adiposity (measured by dual x-ray absorptiometry [DXA] total body fat mass) as a potential confounder. We also considered adiposity and insulin resistance (assessed by the homeostatic model assessment of insulin resistance [HOMA-IR]) at the time of liver outcome assessment as potential mediators (ie, potentially being on the causal pathway between physical activity and NAFLD).

### Eligibility Criteria

For analysis of ultrasound scan liver outcomes, eligible participants had to have valid data for ultrasound scan liver fat and liver stiffness, and a measure of physical activity at mean age 12 and/or 14 years (n = 1292). For analyses of blood-based liver outcomes, eligible participants had to have data for ALT, AST, and GGT and a measure of physical activity at mean age 12 and/or 14 years (n = 2612). As described above, participants’ alcohol consumption was assessed using AUDIT (25). No eligible participants were classified as having harmful levels of alcohol consumption.

### Statistical Analysis

All the analyses were conducted in Stata (StataCorp, College Station, TX). Total physical activity was divided by 100 and MVPA by 15; these values were chosen because they represent realistic intervention targets (32). ALT, AST, GGT, and liver stiffness were positively skewed, and their natural logged values were used in all of the linear regression analyses. Coefficients from regression models including these logged variables as outcomes were back transformed. Associations of total physical activity and MVPA with the binary ultrasound scan liver fat variable were assessed with logistic regression.

Associations of total physical activity and MVPA with liver outcomes were examined using the following regression models: unadjusted; adjusted for, sex, maternal age at delivery, parity, maternal education, head of household, social class, maternal BMI, ethnicity, energy intake at age 11 years, pubertal status at age 11 years, age at physical activity and liver outcome assessments, and the length of time the accelerometer was worn; same as model 2 but additionally adjusted for DXA-determined fat mass, height, and height^2^ at the time physical activity was assessed as a potential confounder; same as model 2 but additionally adjusted for DXA-determined fat mass, height, and height^2^ at the time all of the liver outcomes were assessed as a potential mediator; same as model 4 but additionally adjusted for HOMA-IR at the time all the liver outcomes were assessed as another potential mediator. The inclusion of height and height-squared as covariables in all of the analyses including fat mass is to ensure adjustment for greater relative adiposity, rather than greater fat mass as a result of greater height. Likelihood ratio tests were used to check for sex interactions in all of the models.

#### Dealing With Missing Data and Additional Analyses

Of the 1292 eligible participants with ultrasound scan data and a physical activity measure at ages 12 and/or 14 years, 7% of the participants were missing data for one of the physical activity measures at either age 12 or 14 years. Of the 2612 eligible participants with blood-based liver data and a physical activity measure at ages 12 and/or 14 years, 30% of the participants were missing data for one of the physical activity measures at either age 12 or 14 years. There was also missing data for potential confounders. Web Tables A and B, show the percentage of imputed data for each variable. To minimize selection bias and increase efficiency, multivariate multiple imputation was used to impute missing data for eligible participants. Full details of this procedure are in the online supplement. Details of a series of sensitivity analyses, conducted to verify model assumptions and to test the robustness of our findings, are also provided in the online supplement.

## RESULTS

Characteristics of the ultrasound scan study participants stratified by ultrasound scan–identified liver fat are described in Table 1. The sample consisted of 96% white participants. There was no evidence that associations of total physical activity and MVPA at ages 12 and 14 years with the liver outcomes differed between boys and girls (all interaction *P* values >0.05). Thus, results are presented with sexes combined, and sex is adjusted for in all the models.

Prevalence of ultrasound scan fatty liver was 2.7% in boys (n = 14/524) and 2.1% in girls (n = 16/768). ALT was elevated (>40 U/L) in 4.2% of the boys (n = 53/1249) and 1.8% of the girls (n = 25/1363). Variable distributions were similar among the imputed and observed data sets (Web Tables C and D.

### Associations of Total Physical Activity and MVPA at Mean Ages 12 and 14 Years With the Ultrasound Scan Liver Outcomes at Mean Age 17.8 Years

Confounder and mediator-adjusted associations (ie, models 2–5) of total physical activity and MVPA with the ultrasound scan liver outcomes are presented in Table 2. Unadjusted results are in Web Table E. There was evidence of an inverse association of total physical activity and MVPA at ages 12 and 14 years with the risk of ultrasound scan liver fat in the unadjusted model (model 1, Web Table E and after adjusting for potential confounders (model 2, Table 2). Associations attenuated toward the null after additional adjustment for fat mass at the time of physical activity assessment as a potential confounder (model 3, Table 2), and to a lesser extent after adjustment for fat mass at the time of liver outcome assessment as a potential mediator (model 4, Table 2). Associations did not further attenuate upon additional adjustment of HOMA-IR as a potential mediator (model 5, Table 2). Coefficient magnitudes were greater at age 12 years compared with age 14 years. There were no associations of total physical activity and MVPA with liver stiffness (Table 2).

### Associations of Total Physical Activity and MVPA at Ages 12 and 14 Years With the Blood-Based Liver Outcomes at Mean Age 17.8 Years

Confounder and mediator-adjusted associations (ie, models 2–5) of total physical activity and MVPA with the blood-based liver outcomes are presented in Table 3. Unadjusted results are in Web Table F. After adjusting for potential confounders (model 2, Table 3), there was no evidence of associations of total physical activity or MVPA at 12 or 14 years with ALT. There was evidence for small positive associations of total physical activity and MVPA at age 12 years with AST in all the models. There was also evidence of positive associations of total physical activity and MVPA at age 14 years with AST in the unadjusted model (model 1, Web Table F and the point estimate did not change after adjustment for potential confounders and mediators (models 2–5, Table 3). After adjusting for potential confounders (model 2, Table 3), there was strong evidence of small inverse associations of total physical activity and MVPA at age 12 years with GGT. Point estimates attenuated toward the null after adjusting for fat mass as a potential confounder (model 3, Table 3) or a potential mediator (model 4, Table 3), and associations did not change after additional adjustment for HOMA-IR as a potential mediator (model 5, Table 3).

### Additional Analyses

Results were similar when restricted to participants with complete data for all the variables except confidence intervals were much wider, likely because of the large reduction in sample size (ultrasound scan data set reduced from n = 1292 in the imputed data to n = 506 in the complete case, and the biomarker data set reduced from n = 2612 to n = 1117, Web Tables G and H. The results were also similar when multiple imputation models were restricted to participants with data for physical activity at age 12 years (Web Tables I and J and participants with data for physical activity at age 14 (Web Tables K and L. There was no evidence that the odds of missing physical activity data at age 14 years were associated with physical activity at age 12 years, adjusted for all of the variables included in our multiple imputation models (Web Table M. Results were similar after additional adjustment for AUDIT scores in the year before assessing liver outcomes (Web Tables N and O.

## DISCUSSION

In this study, we assessed prospective associations of objectively measured physical activity at mean ages 12 and 14 years with measures of liver fat at mean age 17.8 years. Greater total physical activity and MVPA, at both ages 12 and 14 years, were prospectively associated with lower risk of ultrasound scan liver fat. These associations with ultrasound scan liver fat attenuated toward the null after adjusting for fat mass as a potential confounder, and to a lesser extent with adjustment for fat mass as a potential mediator. Greater total physical activity and MVPA at age 12 years were also prospectively associated with lower levels of GGT, which is associated with cardiovascular disease (33), diabetes (34), and insulin resistance (35). These associations attenuated toward null after adjusting for fat mass as a potential confounder and as a potential mediator. Associations did not further attenuate upon additional (to fat mass) adjustment of HOMA-IR as a potential mediator. Thus, the effect of physical activity on the liver outcomes may operate through adiposity mediating the relation of physical activity to NAFLD, and/or through greater adiposity leading to reduced physical activity (19,20) and greater NAFLD risk, thus confounding the association, or a combination of both.

Cross-sectional studies have reported inverse associations with ultrasound scan liver fat (12,14,16) and biomarkers, including ALT (11–13) and GGT (11), with some of these studies reporting associations to be independent of adiposity measures (13,16). A small number of studies show no association of physical activity with ultrasound scan or biomarkers of liver fat in children, irrespective of adjustment for adiposity (10,17,18). Randomized controlled trials show beneficial effects of lifestyle interventions on markers of liver fat, including on levels of GGT and ALT providing some support for a possible causal effect (3,36–38). Those trials, however, have all combined physical activity interventions with nutrition programs or antioxidant therapy; thus, it is impossible to assess the relative contribution of physical activity to the observed improvements in NAFLD parameters.

We found no associations of physical activity with ALT in our study. As noted above, in cross-sectional studies of children, greater levels of physical activity have been related to lower ALT levels. Given the prospective association we observed with ultrasound scan fatty liver, which is a more direct measure of liver fat, the lack of association with ALT in our study is difficult to explain but could reflect random sampling variation. We also found that childhood physical activity was not associated with ultrasound scan–assessed liver stiffness. This may reflect earlier physical activity reducing the risk of fat in the liver but not being sufficient to influence fibrosis, if it has become established. Further examination in prospective studies with relevant data is important before making conclusions about these findings.

We found that children with greater levels of physical activity in childhood had higher AST levels in adolescence. AST is abundant in muscle cells, and studies have reported greater physical activity, particularly vigorous activity, to be associated with higher circulating levels possibly because of muscle cell breakdown (39–41). Consistent with this and our findings, a recent cross-sectional study found that adolescents achieving daily recommended time spent in objectively measured MVPA (compared with those not achieving this recommendation) had lower GGT and ALT but higher AST (42).

### Strengths and Limitations

To our knowledge, this is the first study to prospectively assess associations of objectively measured physical activity in childhood with the risk of NAFLD in adolescence. Although power for the binary outcome of ultrasound scan–measured liver fat was limited because of the small number of cases, we looked at a range of related outcomes including ALT. A recent meta-analysis including 49 different studies found ultrasound scan to be reliable and accurate for the detection of moderate-severe hepatic steatosis compared with liver histology (43) but less able to distinguish mild fat infiltration. This means our NAFLD cases are likely to reflect the moderate/severe end of the disease spectrum, and the associations observed between physical activity and ultrasound scan liver fat may be underestimated. The ARFI measure of liver stiffness has been validated in a small number of clinical studies (44,45). Although there was a greater proportion of missing data for the physical activity measure at age 14 years compared with at age 12, results were similar for the complete case analyses and when restricting multiple imputation analyses to those with a measure of physical activity at ages 12 and 14 years (ie, only imputing missing confounder data). Furthermore, we found no evidence that missingness at age 14 years was associated with physical activity at age 12 years. Together, these findings suggest that missing data is unlikely to importantly introduce bias in these associations.

## CONCLUSIONS

We have shown that adolescents who were more active in late childhood have lower fat mass in adolescence, which in turn is related to the lower risk of ultrasound scan fatty liver and lower levels of GGT. If our findings are replicated, they highlight the importance of maintaining healthy levels of physical activity through childhood to prevent the risk of subsequent NAFLD.

## Supplementary Material

Supplemental Digital Content

## Figures and Tables

**TABLE 1:** Characteristics of participants included in the ultrasound scan study (N = 1292)

	Participants without ultrasound scan liver fat (n = 1262)		Participants with ultrasound scan liver fat (n = 30)		
	n with available data	Distribution	n with available data	Distribution	*P* for difference
Median liver stiffness (IQR)	1262	1.18 (1.07, 1.31)	30	1.41 (1.23, 1.98)	<0.001
Median ALT, U/L (IQR)	866	15.5 (12.4, 19.6)	16	20.65 (17.1, 42.7)	<0.001
Median AST, U/L (IQR)	866	19.7 (16.9, 23.3)	16	21.6 18.15 31.75	
				21.6 18.15 31.75	
				21.6 (18.15, 31.75)	0.06
Median GGT, U/L (IQR)	865	16.0 (13.0, 20.0)	16	28.5 (17.0, 41.0)	<0.01
Median CPM at age 12 y (IQR)	1180	564 (464, 694)	28	481 (441, 613)	0.03
Median CPM at age 14 y (IQR)	882	500 (398, 650)	21	420 (397, 524)	0.08
Median MVPA at age 12 y, minutes per day (IQR)	1180	18.4 (11.07, 29.83)	28	14.51 (7.75, 18.57)	0.02
Median MVPA at age 14 y, minutes per day (IQR)	882	19.15 (9.80, 32.0)	21	15.67 (6.0, 22.0)	0.08
Mean maternal age, y (SD)	1220	29.58 (26.58, 32.83)	27	30 (27.58, 33.42)	0.60
Median maternal BMI, kg/m^2^ (IQR)	1109	22.18 (20.47, 24.38)	26	23.50 (21.33, 26.49)	<0.01
Median predicted energy intake at age 12 y, kcal (IQR)	1258	2011 (1911, 2156)	30	2051 (1963, 2234)	0.11
Median fat mass at mean age 12 y, kg (IQR)	1208	10.1 (6.8, 15.0)	29	19.5 (16.1, 27.5)	<0.001
Median fat mass at mean age 14 y, kg (IQR)	1115	12.2 (7.9, 18.1)	26	26.9 (18.1, 30.4)	<0.001
Median fat mass at mean age 17.8 y, kg (IQR)	1224	16.6 (11.3, 23.3)	29	36.9 (28.9, 46.6)	<0.001
Median HOMA-IR at mean age 17.8 y, kg (IQR)	865	1.49 (1.08, 2.09)	16	3.24 (2.24, 4.23)	<0.001
Mean height at mean age 17.8 y, cm (SD)	1234	170.57 (9.40)	30	171.81 (8.98)	0.48
Median age at outcome assessment, mo (IQR)	1262	213 (212, 216)	30	214 (213, 217)	0.16
BMI category	1233		30		
Obese		5.52		53.33	<0.001
Overweight		16.30		30.00	
Normal weight		73.15		10.00	
Underweight		5.03		6.67	
Sex	1262		30		
Males		40.41		46.67	0.49
Females		59.59		53.33	
Parity	1165		26		
0		49.61		34.62	0.30
1		34.59		42.31	
2+		15.79		23.08	
Head of household, social class					
Manual	1134	11.99	24	16.67	0.49
Nonmanual		88.01		83.33	
Mothers’ education	1148		24		
≤O level		51.39		41.67	0.55
A level		27.96		37.50	
Degree or above		20.64		20.83	
Tanner stage for pubic hair development at age 11 y	946		21		
Prepubertal		62.58		76.19	0.65
Pubertal/postpubertal		37.42		23.81	
Alcohol consumption in the year before outcome assessment	1166		29		
Normal		63.72		62.07	0.95
Hazardous		31.73		31.03	
Harmful		4.55		6.90	

When the median and IQR is displayed, a Mann-Whitney *U* test was used to check for differences between those with and without ultrasound scan liver fat. When the mean and SD are displayed, a 2-tailed *t* test was used to check for differences between those with and without ultrasound scan liver fat. The ‘N with available data’ columns relate to the number of participants included in the imputation data sets, who had data for each of the variables included in our analysis. The highest pubertal stage was established by taking the highest Tanner rating for either breast development or pubic hair. If there were missing data for breast development, pubic hair ratings were used when available and vice versa. BMI was categorized as follows: underweight (BMI ≤ 18), normal weight (BMI >18 to ≤25), overweight (BMI >25 to ≤30), and obese (BMI > 30). ALT = alanine aminotransferase; AST = aspartate aminotransferase; BMI = body mass index; CPM = counts per minute; GGT = γ-glutamyl transferase; HOMA-IR = homeostatic model assessment of insulin resistance; IQR = interquartile range; MVPA = moderate to vigorous physical activity; SD = standard deviation.

**TABLE 2:** Associations of physical activity measures at ages 12 and 14 years with USS liver outcomes at mean age 17.8 years in the imputed data (n = 1292)

	Adjusted for confounders^*^		Adjusted for confounders plus fat mass, height, and height^2^ at the time physical activity was assessed^†^		Adjusted for confounders plus fat mass, height, and height^2^ at the time of liver outcome assessment^‡^		Adjusted for confounders plus fat mass, height, height^2^, and HOMA-IR at the time of liver outcome assessment^§^	
USS liver fat								
	OR (95% CI)	*P*	OR (95% CI)	*P*	OR (95% CI)	*P*	OR (95% CI)	*P*
Total physical activity								
12 y	0.71 (0.54–0.94)	0.02	0.82 (0.60–1.12)	0.22	0.76 (0.56–1.03)	0.08	0.73 (0.52–1.01)	0.06
14 y	0.77 (0.58–1.02)	0.07	0.84 (0.61–1.15)	0.28	0.82 (0.60–1.10)	0.19	0.79 (0.57–1.08)	0.14
MVPA								
12 y	0.47 (0.27–0.84)	0.01	0.65 (0.35–1.21)	0.17	0.59 (0.32–1.09)	0.09	0.53 (0.27–1.03)	0.06
14 y	0.63 (0.39–1.04)	0.07	0.72 (0.42–1.26)	0.26	0.70 (0.41–1.19)	0.19	0.67 (0.39–1.13)	0.13

USS liver stiffness								
	% Change (95% CI)	*P*	% Change (95% CI)	*P*	% Change (95% CI)	*P*	% Change (95% CI)	*P*
Total physical activity								
12 y	0 (−1 to 1)	0.96	0 (0–1)	0.35	0 (0–1)	0.67	0 (0–1)	0.66
14 y	0 (−1 to 0)	0.21	0 (−1 to 0)	0.44	0 (−1 to 0)	0.27	0 (−1 to 0)	0.26
MVPA								
12 y	0 (−1 to 1)	0.83	1 (−1 to 2)	0.36	0 (−1 to 1)	0.72	0 (−1 to 1)	0.72
14 y	−1 (−2 to 0)	0.23	0 (−1 to 1)	0.44	−1 (−2 to 0)	0.27	−1 (−2 to 0)	0.26

Coefficients are per increase of 100 CPM (total physical activity) or per 15-minute increase in MVPA. Unadjusted results (model 1) are provided in Table E of the online supplement. BMI = body mass index; CI = confidence interval; CPM = counts per minute; HOMA-IR = homeostatic model assessment of insulin resistance; MVPA = moderate to vigorous physical activity; OR = odds ratio; USS = ultrasound scan.

^*^Model 2: adjusted for mother's age at delivery, parity, sex, ethnicity, mother's education, head of household, social class, mother's BMI, energy intake at age 11 years, pubertal status at age 11 years, age at physical activity assessment, length of time accelerometer was worn (in minutes), and age at the time of liver assessment.

^†^Model 3: adjusted for all of the potential confounders listed above plus fat mass, height, and height^2^ at the time physical activity was assessed.

^‡^Model 4: adjusted for all of the potential confounders listed above plus fat mass, height, and height^2^ at the time of liver outcome assessment.

^§^Model 5: adjusted for all potential confounders listed above plus fat mass, height, height^2^ and HOMA-IR at the time of liver outcome assessment.

**TABLE 3:** Associations of physical activity at ages 12 and 14 years with blood-based liver outcomes at mean age 17.8 years in the imputed data (n = 2612)

	Adjusted for confounders^*^		Adjusted for confounders plus fat mass, height, and height^2^ at the time physical activity was assessed^†^		Adjusted for confounders plus fat mass, height, and height^2^ at the time of liver outcome assessment^‡^		Adjusted for confounders plus fat mass, height, height^2^, and HOMA-IR at the time of liver outcome assessment^§^	
	% Change (95% CI)	*P*	% Change (95% CI)	*P*	% Change (95% CI)	*P*	% Change (95% CI)	*P*
ALT								
Total physical activity								
12 y	0 (−1 to 1)	0.81	1 (0–2)	0.22	1 (0–2)	0.17	1 (0–2)	0.17
14 y	0 (−1 to 1)	0.74	0 (−1 to 1)	0.59	0 (−1 to 1)	0.45	0 (−1 to 1)	0.44
MVPA								
12 y	−1 (−3 to 1)	0.28	0 (−1 to 2)	0.68	1 (−1 to 3)	0.39	1 (−1 to 3)	0.40
14 y	−1 (−3 to 1)	0.31	0 (−2 to 1)	0.75	0 (−2 to 2)	0.97	0 (−2 to 2)	0.98
AST								
Total physical activity								
12 y	1 (0–1)	0.02	1 (0–1)	0.03	1 (0–2)	0.01	1 (0–2)	0.01
14 y	1 (0–1)	0.09	1 (0–1)	0.11	1 (0–1)	0.05	1 (0–1)	0.05
MVPA								
12 y	1 (0–2)	0.05	1 (0–2)	0.05	2 (0–3)	0.01	2 (0–3)	0.01
14 y	1 (0–2)	0.13	1 (0–2)	0.13	1 (0–3)	0.07	1 (0–3)	0.07
GGT								
Total physical activity								
12 y	−1 (−2 to 0)	<0.01	−1 (−2 to 0)	<0.01	0 (−1 to 0)	0.25	0 (−1 to 0)	0.23
14 y	−1 (−2 to 0)	0.21	−1 (−2 to 0)	0.21	0 (−1 to 1)	0.91	0 (−1 to 0)	0.23
MVPA								
12 y	−3 (−4 to −1)	<0.01	−3 (−4 to −1)	<0.01	−1 (−3 to 0)	0.15	0 (−3 to 0)	0.13
14 y	−1 (−2 to 1)	0.31	−1 (−2 to 1)	0.31	0 (−1 to 2)	0.90	0 (−1 to 2)	0.94

Coefficients are per increase of 100 CPM (total physical activity) or per 15-minute increase in MVPA. Unadjusted results (model 1) are provided in Table F of the online supplement. ALT = alanine aminotransferase; AST = aspartate aminotransferase; BMI = body mass index; CI = confidence interval; CPM = counts per minute; GGT = γ-glutamyl transferase; HOMA-IR = homeostatic model assessment of insulin resistance; MVPA = moderate to vigorous physical activity.

^*^Model adjusts for mother's age at delivery, parity, sex, ethnicity, mother's education, head of household, social class, mother's BMI, energy intake at age 11 years, pubertal status at age 11 years, age at physical activity assessment, length of time accelerometer was worn (in minutes), and age at the time of liver assessment.

^†^Model adjusts for all the potential confounders listed above plus fat mass, height, and height^2^ at the time physical activity was assessed.

^‡^Model adjusts for all the potential confounders listed above plus fat mass, height, and height^2^ at the time of liver outcome assessment.

^§^Model adjusts for all the potential confounders listed above plus fat mass, height, height^2^, and HOMA-IR at the time of liver outcome assessment.
