# Supplementary material for: Physical Activity Is Prospectively Associated With Adolescent Nonalcoholic Fatty Liver Disease
Source: J Pediatr Gastroenterol Nutr. 2015 Aug 4;62(1):110–7. doi: 10.1097/MPG.0000000000000904 (PMC4697952; doi:10.1097/MPG.0000000000000904)
Supplement: Supplemental Digital Content [file jpga-62-110-s001.docx]

**Online supplement**

**Assessment of physical activity**

Physical activity was objectively measured at mean ages 12, 14 and 15 years with uniaxial Actigraph accelerometers (Actigraph AM7164 2.2; LLC, Fort Walton Beach, Florida; <http://www.theactigraph.com>),(293) which measure activity with substantially increased precision in comparison to self-report methods.(294) The Actigraph has been validated for the assessment of physical activity in children and adolescents against heart-rate telemetry,(295) indirect calorimetry,(296) and energy expenditure measured by doubly labelled water.(297) Children attending the follow-up clinics at mean ages 12, 14 and 15 years were asked to wear the accelerometer for seven days. The Actigraphs were programmed to begin recording at 5am on the day following the clinic visit and children were asked to start wearing the accelerometer on their right hip on the morning following the clinic visit. Children were asked to wear the Actigraphs during waking hours and to take it off only for showering, bathing, or water sports. They were also asked to record the times at which the Actigraph was put on and taken off and any times (in minutes) that they swam or cycled each day as the Actigraph does not record cycling well and cannot be worn for swimming. Actigraphs were downloaded on to a computer and two variables describing levels and patterns of physical activity were derived: average counts per minute (CPM) and average time spent in moderate to vigorous physical activity (MVPA) in minutes over the valid measurement period. The cut-off point used to define MVPA was a CPM greater than 3600 and this was derived from a calibration study of 246 children, in which Actigraph counts/min were compared with oxygen uptake.(298)

If there were 10 or more minutes of consecutive zeros in any data file, these were deleted as these periods were regarded as times when the monitor was not worn. Any recording day on which the average CPM was less than 150 or more than 3 standard deviations above the mean was also excluded as this level of physical activity was considered behaviourally implausible. In a calibration study on ALSPAC participants,(298) children were asked to walk briskly whilst wearing an Actigraph and CPM ranged from 1816 to 7136. The mean plus 3 standard deviations was 1665 CPM, and it was considered unlikely that a child could sustain this level of intensity for an entire day. Children were also asked to lie still for 5 minutes and then sit still for 5 minutes whilst wearing an Actigraph. Eighty-eight percent of children lay still enough to accrue no counts and 77% sat still enough to accrue no counts. Six children did have from 60 to over 100 CPM for each period of either lying or sitting. It was considered unlikely that many children could maintain a level of average activity below 150 CPM over an entire day. Activity data was considered to be valid if the recording period was for at least 10 hours per day for at least three days. Instruments were calibrated with every battery change which corresponded to intervals of approximately 6 months.

**Assessment of covariables**

Maternal age at delivery of the child and child’s sex were obtained from obstetric records. Questionnaires administered to mothers early in pregnancy asked about ethnicity, parity, educational attainment and pre-pregnancy height and weight. Fathers were also asked to report their height and weight. Parental body mass index (BMI) was calculated as weight in kilograms divided by height in meters squared. Based on questionnaire responses, the highest parental occupation was used to allocate participants to family social class groups using the 1991 British Office of Population and Census Statistics (OPCS) classification. Age was recorded at the time of each physical activity assessment, along with the length of time the accelerometer was worn (in minutes). Energy intake at age 11 was predicted from individual trajectories that have been previously modelled for ALSPAC participants.(1) A previous publication details how energy intake trajectories were modelled and how energy intake at particular ages is calculated using the trajectories. Pubertal stage at the follow-up clinic at mean age 11 years was assessed by a questionnaire in which the respondent was asked to examine line drawings representing the ﬁve Tanner stages for pubic hair and to record which drawing most nearly represented the participant’s current stage of development.(2;3)

At the 17-18 year follow-up visit (i.e. the time of liver outcome assessment), a lunar prodigy narrow fan beam densitometer was used to perform a whole body dual-energy X-ray absorptiometry (DXA) scan, from which total fat mass was measured. Height was measured to the nearest 0.1cm using a Harpenden stadiometer with the participant unshod. Serum insulin was measured with an enzyme linked immunosorbent assay (ELISA, Mercodia, Uppsala, Sweden) that does not cross-react with proinsulin and plasma glucose was measured with an automated assay. From insulin and glucose, we calculated the homeostatic model assessment of insulin resistance (HOMA-IR). Age was recorded at assessment of the liver outcomes.

**Multiple imputation procedure**All participants included in this study had valid data for the USS liver outcomes and the blood-based liver outcomes, however, 83% (n=1087) of eligible participants with USS data, and 80% (n=2121) of eligible participants with blood-based liver outcomes had missing data for CPM, MVPA or potential confounders. To minimise selection bias and increase efficiency, multivariable multiple imputation using chained equations was used to impute missing data for CPM, MVPA and potential confounders for eligible participants. Regression switching was used in Stata, as described by Royston.(4) Twenty cycles of regression switching were carried out and 20 imputation datasets were generated.

The multiple imputation was carried out separately for males and females and the two sets of datasets were appended together to allow us to assess interactions in the multiple imputation datasets. Tables A and B below detail each variable included in the imputation model (liver outcome variables, physical activity variables, potential confounding variables and possible predictors of the missing data), the type of variable (i.e. continuous or categorical) and the type of regression model used to predict missing data in this variable. The multiple multivariable imputation approach creates a specified number of copies of the data (in our case, 20 copies) in which missing values are imputed by chained equations, with an appropriate level of randomness. The main results presented in this paper on the multiple imputation datasets are obtained by averaging the results from each of these 20 datasets using Rubin’s rules. In this procedure, the standard errors for any regression coefficients (used to calculate p-values and 95% confidence intervals) take account of the uncertainty in the imputations as well as uncertainty in the estimate. There was no substantial collinearity between variables included in the multiple imputation models. The analyses based on these multivariable imputations all include data from the 1292 eligible participants in the USS dataset and the 2612 participants in the blood-based liver outcomes dataset. We also repeated analyses including only those participants with complete data on all variables used in our analyses, i.e. with no missing data (n=506 in the USS dataset and n=1117 in the blood-based liver outcome dataset). Results from the complete case analyses were similar to those found based on the multiple imputation datasets except they had wider confidence intervals due to the reduction in sample size (Tables E and F below).

**Additional analyses**

Analyses were repeated for participants with complete data for all variables included in the analysis (i.e. with no missing data; n=497 (38%) in the USS dataset and n=1093 (42%) in the blood-based liver outcome dataset). To further check our results are robust to assumptions about missing data, multiple imputations were repeated after restricting to (i) participants with a physical activity measure at age 12 (n=1208 (93%) for USS outcomes dataset and n=2393 (92%) for blood-based outcomes dataset) and (ii) participants with a physical activity measure at age 14 (n=903 (70%) for USS outcomes dataset and n=2000 (77%) for blood-based outcomes dataset), thereby reducing the amount of missing data being imputed for each age (i.e only imputing missing data for potential confounders, not the physical activity measures). We also checked whether the probability of missing physical activity data at age 14 was related to physical activity level at 12 years, conditional on all other variables included in our multiple imputation models. We conducted a sensitivity analysis adjusting for AUDIT scores from the follow-up clinics at mean ages 16.7y and 17.8y.

**Table A: Variables used in multivariable multiple imputation models for the eligible male (n=524) and female (n=768) participants in the USS sample (total n=1292)**

| **Variable** | **Type of variable** | **Regression model used to predict missing data in this variable** | **How variable entered when used to predict missing in other variables** |
| --- | --- | --- | --- |
| **Liver outcome variables** | | | |
| Liver fat at mean age 17.8 | Binary | N/A no missing data | Binary |
| Liver stiffness at mean age 17.8 | Continuous | N/A no missing data | Continuous |
| **Physical activity variables** | | | |
| CPM at age 12 | Continuous | Linear regression | Continuous |
| MVPA at age 12 | Continuous | Linear regression | Continuous |
| CPM at age 14 | Continuous | Linear regression | Continuous |
| MVPA at age 14 | Continuous | Linear regression | Continuous |
| **Covariables for regression analyses** | | | |
| Age at outcome assessment | Continuous | N/A no missing data | Continuous |
| Age at 12 year assessment | Continuous | Linear regression | Continuous |
| Age at 14 year assessment | Continuous | Linear regression | Continuous |
| Minutes wearing accelerometer at 12 year assessment | Continuous | Linear regression | Continuous |
| Minutes wearing accelerometer at 14 year assessment | Continuous | Linear regression | Continuous |
| Predicted energy intake at age 11 | Continuous | N/A no missing data | Continuous |
| Ethnicity | Binary | Multinomial logistic regression | Binary |
| Parity | Categorical (3 categories) | Ordinal logistic regression | 2 indicator variables |
| Tanner stage for pubic hair | Binary | Ordinal logistic regression | Binary |
| Maternal age at delivery (months) | Continuous | Linear regression | Continuous |
| Maternal education | Categorical (3 categories) | Ordinal logistic regression | 2 indicator variables |
| Head of household social class | Binary | Ordinal logistic regression | Binary |
| Maternal pre-pregnancy BMI | Continuous | Linear regression | Continuous |
| Maternal pre-pregnancy smoking | Categorical (3 categories) | Ordinal logistic regression | 2 indicator variables |
| Maternal pre-pregnancy alcohol consumption | Categorical (3 categories) | Ordinal logistic regression | 2 indicator variables |
| Fat mass at 12 year assessment | Continuous | Linear regression | Continuous |
| Fat mass at 14 year assessment | Continuous | Linear regression | Continuous |
| Fat mass at outcome assessment | Continuous | Linear regression | Continuous |
| Height at outcome assessment | Continuous | Linear regression | Continuous |
| Height^2^ at outcome assessment | Continuous | Linear regression | Continuous |
| AUDIT score at age 16 | Continuous | Linear regression | Continuous |
| AUDIT score at age 17 | Continuous | Linear regression | Continuous |
| **Other possible predictors of missing data** | | | |
| Liver volume at mean age 17.8 | Continuous | Linear regression | Continuous |
| CPM at age 15 | Continuous | Linear regression | Continuous |
| MVPA at age 15 | Continuous | Linear regression | Continuous |
| Age at 15 year assessment | Continuous | Linear regression | Continuous |
| Minutes wearing accelerometer at 15 year assessment | Continuous | Linear regression | Continuous |
| Height at 12 year assessment | Continuous | Linear regression | Continuous |
| Weight at 12 year assessment | Continuous | Linear regression | Continuous |
| Height at 14 year assessment | Continuous | Linear regression | Continuous |
| Weight at 14 year assessment | Continuous | Linear regression | Continuous |
| Height at 15 year assessment | Continuous | Linear regression | Continuous |
| Weight at 15 year assessment | Continuous | Linear regression | Continuous |
| HOMA-IR at age 17 | Continuous | Linear regression | Continuous |

**Table B: Variables used in multivariable multiple imputation models for the eligible male (n=768) and female (n=1363) participants in the biomarker sample (total n=2612)**

| **Variable** | **Type of variable** | **Regression model used to predict missing data in this variable** | **How variable entered when used to predict missing in other variables** |
| --- | --- | --- | --- |
| **Liver outcome variables** | | | |
| ALT at mean age 17.8 | Continuous | Linear regression | Continuous |
| AST at mean age 17.8 | Continuous | Linear regression | Continuous |
| GGT at mean age 17.8 | | | |
| **Physical activity variables** | | | |
| CPM at age 12 | Continuous | Linear regression | Continuous |
| MVPA at age 12 | Continuous | Linear regression | Continuous |
| CPM at age 14 | Continuous | Linear regression | Continuous |
| MVPA at age 14 | Continuous | Linear regression | Continuous |
| **Covariables for regression analyses** | | | |
| Age at outcome assessment | Continuous | N/A no missing data | Continuous |
| Age at 12 year assessment | Continuous | Linear regression | Continuous |
| Age at 14 year assessment | Continuous | Linear regression | Continuous |
| Minutes wearing accelerometer at 12 year assessment | Continuous | Linear regression | Continuous |
| Minutes wearing accelerometer at 14 year assessment | Continuous | Linear regression | Continuous |
| Predicted energy intake at age 11 | Continuous | N/A no missing data | Continuous |
| Ethnicity | Binary | Multinomial logistic regression | Binary |
| Parity | Categorical (3 categories) | Ordinal logistic regression | 2 indicator variables |
| Tanner stage for pubic hair | Binary | Ordinal logistic regression | Binary |
| Maternal age at delivery (months) | Continuous | Linear regression | Continuous |
| Maternal education | Categorical (3 categories) | Ordinal logistic regression | 2 indicator variables |
| Head of household social class | Binary | Ordinal logistic regression | Binary |
| Maternal pre-pregnancy BMI | Continuous | Linear regression | Continuous |
| Maternal pre-pregnancy smoking | Categorical (3 categories) | Ordinal logistic regression | 2 indicator variables |
| Maternal pre-pregnancy alcohol consumption | Categorical (3 categories) | Ordinal logistic regression | 2 indicator variables |
| Fat mass at 12 year assessment | Continuous | Linear regression | Continuous |
| Fat mass at 14 year assessment | Continuous | Linear regression | Continuous |
| Fat mass at outcome assessment | Continuous | Linear regression | Continuous |
| Height at outcome assessment | Continuous | Linear regression | Continuous |
| Height^2^ at outcome assessment | Continuous | Linear regression | Continuous |
| AUDIT score at age 16 | Continuous | Linear regression | Continuous |
| AUDIT score at age 17 | Continuous | Linear regression | Continuous |
| **Other possible predictors of missing data** | | | |
| Haptoglobin at mean age 17.8 | Continuous | Linear regression | Continuous |
| CPM at age 15 | Continuous | Linear regression | Continuous |
| MVPA at age 15 | Continuous | Linear regression | Continuous |
| Age at 15 year assessment | Continuous | Linear regression | Continuous |
| Minutes wearing accelerometer at 15 year assessment | Continuous | Linear regression | Continuous |
| Height at 12 year assessment | Continuous | Linear regression | Continuous |
| Weight at 12 year assessment | Continuous | Linear regression | Continuous |
| Height at 14 year assessment | Continuous | Linear regression | Continuous |
| Weight at 14 year assessment | Continuous | Linear regression | Continuous |
| Height at 15 year assessment | Continuous | Linear regression | Continuous |
| Weight at 15 year assessment | Continuous | Linear regression | Continuous |
| HOMA-IR at age 17 | Continuous | Linear regression | Continuous |

**Table C: Distribution of variables in the observed (with no missing) and imputation datasets for USS measures**

| **Imputed Variables** | | **Distribution variables in the observed USS dataset (with no missing) n=497** | **Distribution variables in the imputation dataset for eligible participants with USS data n=1292** | |
| --- | --- | --- | --- | --- |
| **Continuous variables** | | **Mean (SE)/Median (IQR)** | **% Imputed data** | **Mean (SE)/Median (IQR)** |
| Median USS liver stiffness, m/s (IQR) | | 1.2 (1.1, 1.3) | 0 | 1.2 (1.1, 1.3) |
| Median counts per minute at age 12 (IQR) | | 549 (447, 677) | 7 | 562 (461, 690) |
| Median counts per minute at age 14 (IQR) | | 484 (384, 613) | 30 | 500 (398, 648) |
| Median MVPA at age 12, minutes per day (IQR) | | 17 (11, 28) | 7 | 18 (11, 30) |
| Median MVPA at age 14, minutes per day (IQR) | | 19 (9, 31) | 30 | 19 (10, 32) |
| Mean maternal age, years (SE) | | 30.3 (0.2) | 3 | 29.9 (0.03) |
| Median maternal BMI, kg/m^2^ (IQR) | | 22.0 (20.3, 24.1) | 12 | 22.0 (20.5, 24.5) |
| Median energy intake at age 12, kcal (IQR) | | 1995 (1897, 2140) | 0.3 | 2011 (1911, 2156) |
| Median fat mass at mean age 12, kg (IQR) | | 10.1 (6.8, 15.6) | 4 | 10.3 (6.8, 15.2) |
| Median fat mass at mean 14, kg (IQR) | | 12.3 (8.3, 18.0) | 11 | 12.3 (8.0, 18.4) |
| Median fat mass at mean age 17.8, kg (IQR) | | 17.1 (12.2, 23.3) | 3 | 16.7 (11.4, 23.8) |
| Mean height at mean age 17.8, cm (SE) | | 170.2 (0.41) | 2 | 170.6 (0.1) |
| Median age at outcome assessment, months IQR) | | 213 (212, 216) | 0 | 214 (212, 216) |
| **Categorical variables** | | **Percent** |  | **Percent** |
| USS liver fat | Yes | 97.8 | 0 | 97.7 |
|  | No | 2.2 |  | 2.3 |
| Sex | Male | 36.4 | 0 | 40.6 |
|  | Female | 63.6 |  | 59.4 |
| Ethnicity | White | 97.6 | 7 | 96.2 |
|  | Non-white | 2.4 |  | 3.8 |
| Parity | 0 | 53.5 | 8 | 49.4 |
|  | 1 | 33.6 |  | 34.7 |
|  | 2+ | 12.9 |  | 15.9 |
| Head of household social class | Manual | 9.9 | 10 | 12.2 |
|  | Non-manual | 90.1 |  | 87.5 |
| Mother’s Education | <=O level | 48.4 | 9 | 51.6 |
|  | A level | 29.3 |  | 28.0 |
|  | Degree or above | 22.3 |  | 20.4 |
| Tanner stage for pubic hair development at age 11 years | Pre-pubertal | 62.4 | 25 | 63.1 |
|  | Pubertal/post-pubertal | 37.6 |  | 36.9 |
| Alcohol consumption in the year prior to outcome assessment | Hazardous | 30.2 | 8 | 32.3 |
|  | Harmful | 2.9 |  | 4.1 |

SE- Standard Error

**Table D: Distribution of variables in the observed (with no missing) and imputation datasets for blood-based liver outcomes**

| **Imputed Variables** | | **Distribution variables in the observed blood-based liver outcomes dataset (with no missing) n=1093** | **Distribution variables in the imputation dataset for eligible participants with blood-based outcome data n=2612** | |
| --- | --- | --- | --- | --- |
| **Continuous variables** | | **Mean (SE)/Median (IQR)** | **% Imputed data** | **Mean (SE)/Median (IQR)** |
| Median ALT, U/L (median, IQR) | | 15.1 (12.1, 19.3) | 0 | 14.9 (11.9, 19.4) |
| Median AST, U/L (median, IQR) | | 19.6 (16.9, 23.2) | 0 | 19.5 (16.7, 23.2) |
| Median GGT, U/L (median, IQR) | | 16.0 (13.0, 20.0) | 0 | 16.0 (13.0, 21.0) |
| Median counts per minute at age 12 (IQR) | | 567 (471, 696) | 8 | 575 (474, 701) |
| Median counts per minute at age 14 (IQR) | | 505 (403, 631) | 23 | 516 (409, 651) |
| Median MVPA at age 12, minutes per day (IQR) | | 19 (12, 30) | 8 | 20 (12, 31) |
| Median MVPA at age 14, minutes per day (IQR) | | 21 (12, 33) | 23 | 21 (12, 33) |
| Mean maternal age, years (mean, SE) | | 30.2 (0.1) | 4 | 29.9 (0.2) |
| Median maternal BMI, kg/m^2^ (median, IQR) | | 21.9 (20.4, 23.9) | 12 | 22.2 (20.5, 24.2) |
| Median fat mass at mean age 12, kg (IQR) | | 9.7 (6.6, 13.9) | 4 | 9.9 (6.8, 14.8) |
| Median fat mass at mean 14, kg (IQR) | | 11.7 (7.5, 17.0) | 8 | 11.9 (7.6, 17.5) |
| Median fat mass at mean age 17.8y, kg (median, IQR) | | 15.9 (10.7, 21.9) | 3 | 16.0 (10.3, 22.7) |
| Mean height at mean age 17.8y, cm (mean, SE) | | 171.8 (0.3) | 2 | 172.0 (0.2) |
| Median age at outcome assessment, months (median, IQR) | | 212 (210, 214) | 0 | 212 (211, 214) |
| **Categorical variables** | | **Percent** |  | **Percent** |
| Sex | Male | 45.3 | 0 | 47.8 |
|  | Female | 54.7 |  | 52.2 |
| Ethnicity | White | 96.8 | 8 | 96.1 |
|  | Non-white | 3.2 |  | 3.9 |
| Parity | 0 | 50.0 | 7 | 48.4 |
|  | 1 | 35.4 |  | 34.9 |
|  | 2+ | 14.6 |  | 16.7 |
| Head of household social class | Manual | 90.6 | 9 | 11.7 |
|  | Non-manual | 9.4 |  | 88.3 |
| Mother’s Education | <=O level | 43.7 | 9 | 47.5 |
|  | A level | 32.5 |  | 30.9 |
|  | Degree or above | 23.8 |  | 21.6 |
| Tanner stage for pubic hair development at age 11 years | Pre-pubertal | 63.3 | 25 | 63.8 |
|  | Pubertal/post-pubertal | 36.7 |  | 36.2 |
| Alcohol consumption in the year prior to outcome assessment* | Hazardous | 33.2 | 5 | 34.6 |
|  | Harmful | 3.1 |  | 3.4 |

SE- Standard Error

**Table E: Unadjusted associations of physical activity measures at ages 12 and 14 years with USS liver outcomes at mean age 17.8 years in the imputed data (n=1292)**

|  | **Model 1- Unadjusted** | | |  |
| --- | --- | --- | --- | --- |
| **USS Liver Fat** | | | | |
|  | **OR** | **95% CI** | **p** |  |
| **Total physical activity** |  |  |  |  |
| 12 years | 0.76 | (0.58,0.98) | 0.03 |  |
| 14 years | 0.79 | (0.60,1.04) | 0.10 |  |
| **MVPA** |  |  |  |  |
| 12 years | 0.55 | (0.32,0.94) | 0.03 |  |
| 14 years | 0.63 | (0.39,1.02) | 0.06 |  |
| **USS Liver Stiffness** | | | | |
|  | **% Change** | **95% CI** | **p** |  |
| **Total physical activity** |  |  |  |  |
| 12 years | 0% | (-1%,1%) | 0.87 |  |
| 14 years | 0% | (-1%,0%) | 0.18 |  |
| **MVPA** |  |  |  |  |
| 12 years | 0% | (-1%,1%) | 0.65 |  |
| 14 years | -1% | (-2%,0%) | 0.15 |  |

Coefficients are per increase of 100 counts per minute (total physical activity) or per 15 minute increase in MVPA.

**Table F: Unadjusted associations of physical activity at ages 12 and 14 years with blood-based liver outcomes at mean age 17.8 years in the imputed data (n=2612)**

|  | **Model 1-Unadjusted** | | |  |
| --- | --- | --- | --- | --- |
| **ALT** | | | | |
|  | **% Change** | **95% CI** | **p** |  |
| **Total physical activity** |  |  |  |  |
| 12 years | 1% | (0%,2%) | 0.05 |  |
| 14 years | 1% | (0%,2%) | 0.03 |  |
| **MVPA** |  |  |  |  |
| 12 years | 1% | (-1%,3%) | 0.20 |  |
| 14 years | 1% | (-1%,3%) | 0.20 |  |
| **AST** | | | | |
|  | **% Change** | **95% CI** | **p** |  |
| **Total physical activity** |  |  |  |  |
| 12 years | 2% | (1%,2%) | <0.01 |  |
| 14 years | 2% | (1%,2%) | <0.01 |  |
| **MVPA** |  |  |  |  |
| 12 years | 3% | (2%,4%) | <0.01 |  |
| 14 years | 3% | (1%,4%) | <0.01 |  |
| **GGT** | | | | |
|  | **% Change** | **95% CI** | **p** |  |
| **Total physical activity** |  |  |  |  |
| 12 years | 0% | (-1%,1%) | 0.46 |  |
| 14 years | 1% | (0%,2%) | 0.02 |  |
| **MVPA** |  |  |  |  |
| 12 years | 1% | (-1%,2%) | 0.42 |  |
| 14 years | 2% | (0%,3%) | 0.02 |  |

Coefficients are per increase of 100 counts per minute (total physical activity) or per 15 minute increase in MVPA.

**Table G: Associations of CPM at age 12 and 14 years with USS liver outcomes at mean age 17.8 years in the complete case dataset (i.e with no missing data, n=497)**

|  | **Model 1** | | |  | **Model 2** | | |  |  | **Model 3** |  |  | **Model 4** | | |  |
| --- | --- | --- | --- | --- | --- | --- | --- | --- | --- | --- | --- | --- | --- | --- | --- | --- |
| **USS Liver Fat** | | | | | | | | | | | | | | | | |
|  | **OR** | **95% CI** | **p** |  | **OR** | **95% CI** | **p** |  | **OR** | **95% CI** | **p** |  | **OR** | **95% CI** | **p** |  |
| **CPM** |  |  |  |  |  |  |  |  |  |  |  |  |  |  |  |  |
| 12 years | 0.67 | (0.42,1.07) | 0.09 |  | 0.60 | (0.35,1.01) | 0.06 |  | 0.76 | (0.42,1.38) | 0.37 |  | 0.63 | (0.34,1.17) | 0.14 |  |
| 14 years | 0.84 | (0.57,1.23) | 0.36 |  | 0.86 | (0.57,1.29) | 0.46 |  | 1.03 | (0.64,1.65) | 0.92 |  | 0.95 | (0.59,1.52) | 0.82 |  |
| **MVPA** |  |  |  |  |  |  |  |  |  |  |  |  |  |  |  |  |
| 12 years | 0.42 | (0.15,1.16) | 0.09 |  | 0.29 | (0.09,0.95) | 0.04 |  | 0.45 | (0.12,1.71) | 0.24 |  | 0.27 | (0.06,1.21) | 0.09 |  |
| 14 years | 0.66 | (0.32,1.33) | 0.24 |  | 0.63 | (0.29,1.37) | 0.24 |  | 0.82 | (0.32,2.06) | 0.67 |  | 0.64 | (0.24,1.67) | 0.36 |  |
| **USS Liver Stiffness** | | | | | | | | | | | | | | | | |
|  | **% Change** | **95% CI** | **p** |  | **% Change** | **95% CI** | **p** |  | **% Change** | **95% CI** | **p** |  | **% Change** | **95% CI** | **p** |  |
| **CPM** |  |  |  |  |  |  |  |  |  |  |  |  |  |  |  |  |
| 12 years | 1% | (0%,1%) | 0.25 |  | 0% | (-1%,1%) | 0.35 |  | 0% | (-1%,2%) | 0.33 |  | 0% | (-1%,1%) | 0.38 |  |
| 14 years | 0% | (-1%,1%) | 0.62 |  | 0% | (-1%,1%) | 0.69 |  | 0% | (-1%,1%) | 0.62 |  | 0% | (-1%,1%) | 0.69 |  |
| **MVPA** |  |  |  |  |  |  |  |  |  |  |  |  |  |  |  |  |
| 12 years | 1% | (0%,3%) | 0.17 |  | 1% | (-1%,3%) | 0.32 |  | 1% | (-1%,3%) | 0.18 |  | 1% | (-1%,3%) | 0.24 |  |
| 14 years | 0% | (-1%,2%) | 0.57 |  | 0% | (-1%,2%) | 0.75 |  | 0% | (-1%,2%) | 0.68 |  | 0% | (-1%,2%) | 0.76 |  |
| Coefficients are per increase of 100 CPM or per 15 minute increase in MVPA. Model 1 is unadjusted. Model 2 adjusts for mother’s age at delivery, parity, sex, ethnicity, mother’s education, head of household social class, mother’s BMI, energy intake at age 11 years, pubertal status at age 11, age at physical activity assessment, length of time accelerometer was worn (in minutes) and age at the time of liver assessment. Model 3 is the same as model 2 but additionally adjusts for fat mass, height and height^2^ at the time physical activity was assessed. Model 4 is the same as model 2 but additionally adjusts for fat mass, height and height^2^ at the time of liver outcome assessment. | | | | | | | | | | | | | | | | |

**Table H: Associations of CPM and MVPA at ages 12 and 14 years with the blood-based liver outcomes at mean age 17.8 years in the complete case dataset (i.e. with no missing data, n=1093)**

|  | **Model 1** | | |  | **Model 2** | | |  | **Model 3** | | |  | **Model 4** | | |
| --- | --- | --- | --- | --- | --- | --- | --- | --- | --- | --- | --- | --- | --- | --- | --- |
| **ALT** | | | | | | | | | | | | | | | |
|  | **% Change** | **95% CI** | **p** |  | **% Change** | **95% CI** | **p** |  | **% Change** | **95% CI** | **p** |  | **% Change** | **95% CI** | **p** |
| **CPM** |  |  |  |  |  |  |  |  |  |  |  |  |  |  |  |
| 12 years | 1% | (-1%,2%) | 0.25 |  | -1% | (-2%,1%) | 0.49 |  | 0% | (-1%,1%) | 0.96 |  | 0% | (-1%,2%) | 0.64 |
| 14 years | 2% | (0%,3%) | 0.02 |  | 0% | (-1%,2%) | 0.78 |  | 1% | (-1%,2%) | 0.38 |  | 1% | (-1%,2%) | 0.26 |
| **MVPA** |  |  |  |  |  |  |  |  |  |  |  |  |  |  |  |
| 12 years | 1% | (-1%,4%) | 0.29 |  | -1% | (-4%,1%) | 0.33 |  | 0% | (-3%,2%) | 0.86 |  | 1% | (-2%,3%) | 0.61 |
| 14 years | 2% | (-1%,4%) | 0.16 |  | -1% | (-3%,1%) | 0.52 |  | 0% | (-2%,2%) | 0.88 |  | 0% | (-2%,2%) | 0.88 |
| **AST** | | | | | | | | | | | | | | | |
|  | **% Change** | **95% CI** | **p** |  | **% Change** | **95% CI** | **p** |  | **% Change** | **95% CI** | **p** |  | **% Change** | **95% CI** | **% Change** |
| **CPM** |  |  |  |  |  |  |  |  |  |  |  |  |  |  |  |
| 12 years | 2% | (1%,3%) | <0.01 |  | 1% | (0%,2%) | 0.28 |  | 0% | (-1%,1%) | 0.61 |  | 1% | (0%,2%) | 0.21 |
| 14 years | 1% | (0%,2%) | <0.01 |  | 0% | (-1%,1%) | 0.76 |  | 0% | (-1%,1%) | 0.96 |  | 0% | (-1%,1%) | 0.71 |
| **MVPA** |  |  |  |  |  |  |  |  |  |  |  |  |  |  |  |
| 12 years | 4% | (2%,5%) | <0.01 |  | 1% | (0%,3%) | 0.13 |  | 1% | (-1%,3%) | 0.26 |  | 2% | (0%,3%) | 0.07 |
| 14 years | 2% | (0%,3%) | 0.01 |  | 0% | (-2%,1%) | 0.91 |  | 0% | (-2%,1%) | 0.75 |  | 0% | (-2%,2%) | 0.98 |
| **GGT** | | | | | | | | | | | | | | | |
|  | **% Change** | **95% CI** | **p** |  | **% Change** | **95% CI** | **p** |  | **% Change** | **95% CI** | **p** |  | **% Change** | **95% CI** | **% Change** |
| **CPM** |  |  |  |  |  |  |  |  |  |  |  |  |  |  |  |
| 12 years | 0% | (-1%,2%) | 0.65 |  | -1% | (-3%,0%) | 0.03 |  | -1% | (-2%,1%) | 0.28 |  | -1% | (-2%,1%) | 0.39 |
| 14 years | 1% | (0%,2%) | 0.15 |  | -1% | (-2%,0%) | 0.18 |  | 0% | (-2%,1%) | 0.61 |  | 0% | (-1%,1%) | 0.77 |
| **MVPA** |  |  |  |  |  |  |  |  |  |  |  |  |  |  |  |
| 12 years | 1% | (-2%,3%) | 0.65 |  | -3% | (-5%,-1%) | 0.01 |  | -2% | (-4%,1%) | 0.14 |  | -1% | (-3%,1%) | 0.37 |
| 14 years | 2% | (0%,4%) | 0.06 |  | -1% | (-3%,1%) | 0.48 |  | 0% | (-2%,2%) | 0.89 |  | 0% | (-2%,2%) | 0.75 |
| Coefficients are per increase of 100 CPM or per 15 minute increase in MVPA. Model 1 is unadjusted. Model 2 adjusts for mother’s age at delivery, parity, sex, ethnicity, mother’s education, head of household social class, mother’s BMI, energy intake at age 11 years, pubertal status at age 11, age at physical activity assessment, length of time accelerometer was worn (in minutes) and age at the time of liver assessment. Model 3 is the same as model 2 but additionally adjusts for fat mass, height and height^2^ at the time physical activity was assessed. Model 4 is the same as model 2 but additionally adjusts for fat mass, height and height^2^ at the time of liver outcome assessment. | | | | | | | | | | | | | | | |

**Table I: Associations of CPM and MVPA at age 12 years with USS liver outcomes at mean age 17.8 years in the imputed data restricted to participants with physical activity data at age 12 (n=1208)**

|  | **Model 1** | | |  | **Model 2** | | |  | **Model 3** | | |  |  | **Model 4** | | |
| --- | --- | --- | --- | --- | --- | --- | --- | --- | --- | --- | --- | --- | --- | --- | --- | --- |
| **USS Liver Fat** | | | | | | | | | | | | | | | | |
|  | **OR** | **95% CI** | **p** |  | **OR** | **95% CI** | **p** |  | **OR** | **95% CI** | **p** |  |  | **p** | **95% CI** | **p** |
| **CPM** |  |  |  |  |  |  |  |  |  |  |  |  |  |  |  |  |
| 12 years | 0.75 | (0.57,0.97) | 0.03 |  | 0.70 | (0.52,0.93) | 0.01 |  | 0.81 | (0.59,1.12) | 0.20 |  |  | 0.74 | (0.54,1.02) | 0.07 |
| **MVPA** |  |  |  |  |  |  |  |  |  |  |  |  |  |  |  |  |
| 12 years | 0.52 | (0.30,0.90) | 0.02 |  | 0.43 | (0.24,0.78) | 0.01 |  | 0.61 | (0.32,1.15) | 0.13 |  |  | 0.55 | (0.29,1.04) | 0.07 |
| **USS Liver Stiffness** | | | | | | | | | | | | | | | | |
|  | **% Change** | **95% CI** | **p** |  | **% Change** | **95% CI** | **p** |  | **% Change** | **95% CI** | **p** |  |  | **% Change** | **95% CI** | **p** |
| **CPM** |  |  |  |  |  |  |  |  |  |  |  |  |  |  |  |  |
| 12 years | 0% | (-1%,1%) | 0.96 |  | 0% | (-1%,1%) | 0.88 |  | 0% | (0%,1%) | 0.30 |  |  | 0% | (0%,1%) | 0.56 |
| **MVPA** |  |  |  |  |  |  |  |  |  |  |  |  |  |  |  |  |
| 12 years | 0% | (-1%,1%) | 0.71 |  | 0% | (-1%,1%) | 0.92 |  | 1% | (-1%,2%) | 0.30 |  |  | 0% | (-1%,2%) | 0.60 |
| Coefficients are per increase of 100 CPM or per 15 minute increase in MVPA. Model 1 is unadjusted. Model 2 adjusts for mother’s age at delivery, parity, sex, ethnicity, mother’s education, head of household social class, mother’s BMI, energy intake at age 11 years, pubertal status at age 11, age at physical activity assessment, length of time accelerometer was worn (in minutes) and age at the time of liver assessment. Model 3 is the same as model 2 but additionally adjusts for fat mass, height and height^2^ at the time physical activity was assessed. Model 4 is the same as model 2 but additionally adjusts for fat mass, height and height^2^ at the time of liver outcome assessment. | | | | | | | | | | | | | | | | |

**Table J: Associations of CPM and MVPA at age 12 years with blood-based liver outcomes at mean age 17.8 years in the imputed data restricted to participants with physical activity data at age 12 (n=2393)**

|  | **Model 1** | | |  | **Model 2** | | |  | **Model 3** | | |  | **Model 4** | | |
| --- | --- | --- | --- | --- | --- | --- | --- | --- | --- | --- | --- | --- | --- | --- | --- |
| **ALT** | | | | | | | | | | | | | | | |
|  | **% Change** | **95% CI** | **p** |  | **% Change** | **95% CI** | **p** |  | **% Change** | **95% CI** | **p** |  | **% Change** | **95% CI** | **p** |
| **CPM** |  |  |  |  |  |  |  |  |  |  |  |  |  |  |  |
| 12 years | 1% | (0%,2%) | 0.04 |  | 0% | (-1%,1%) | 0.78 |  | 1% | (-1%,2%) | 0.32 |  | 1% | (0%,2%) | 0.15 |
| **MVPA** |  |  |  |  |  |  |  |  |  |  |  |  |  |  |  |
| 12 years | 1% | (0%,3%) | 0.14 |  | -1% | (-3%,1%) | 0.25 |  | 0% | (-2%,2%) | 0.73 |  | 1% | (-1%,3%) | 0.36 |
| **AST** | | | | | | | | | | | | | | | |
|  | **% Change** | **95% CI** | **p** |  | **% Change** | **95% CI** | **p** |  | **% Change** | **95% CI** | **p** |  | **% Change** | **95% CI** | **p** |
| **CPM** |  |  |  |  |  |  |  |  |  |  |  |  |  |  |  |
| 12 years | 2% | (1%,3%) | <0.01 |  | 1% | (0%,1%) | 0.03 |  | 1% | (0%,1%) | 0.05 |  | 1% | (0%,2%) | 0.01 |
| **MVPA** |  |  |  |  |  |  |  |  |  |  |  |  |  |  |  |
| 12 years | 3% | (2%,5%) | <0.01 |  | 1% | (0%,2%) | 0.04 |  | 1% | (0%,2%) | 0.06 |  | 2% | (0%,3%) | 0.01 |
| **GGT** | | | | | | | | | | | | | | | |
|  | **% Change** | **95% CI** | **p** |  | **% Change** | **95% CI** | **p** |  | **% Change** | **95% CI** | **p** |  | **% Change** | **95% CI** | **p** |
| **CPM** |  |  |  |  |  |  |  |  |  |  |  |  |  |  |  |
| 12 years | 0% | (-1%,1%) | 0.49 |  | -1% | (-2%,0%) | <0.01 |  | -1% | (-1%,0%) | 0.19 |  | -1% | (-1%,0%) | 0.21 |
| **MVPA** |  |  |  |  |  |  |  |  |  |  |  |  |  |  |  |
| 12 years | 1% | (-1%,2%) | 0.41 |  | -3% | (-4%,-1%) | <0.01 |  | -2% | (-3%,0%) | 0.06 |  | -1% | (-3%,0%) | 0.12 |
| Coefficients are per increase of 100 CPM or per 15 minute increase in MVPA. Model 1 is unadjusted. Model 2 adjusts for mother’s age at delivery, parity, sex, ethnicity, mother’s education, head of household social class, mother’s BMI, energy intake at age 11 years, pubertal status at age 11, age at physical activity assessment, length of time accelerometer was worn (in minutes) and age at the time of liver assessment. Model 3 is the same as model 2 but additionally adjusts for fat mass, height and height^2^ at the time physical activity was assessed. Model 4 is the same as model 2 but additionally adjusts for fat mass, height and height^2^ at the time of liver outcome assessment. | | | | | | | | | | | | | | | |

**Table K: Associations of CPM and MVPA at age 14 years with USS liver outcomes at mean age 17.8 years in the imputed data restricted to participants with physical activity data at age 14 (n=903)**

|  | **Model 1** | | |  | **Model 2** | | |  | **Model 3** | | |  | **Model 4** | | |  |
| --- | --- | --- | --- | --- | --- | --- | --- | --- | --- | --- | --- | --- | --- | --- | --- | --- |
|  | **USS Liver Fat** | | | | | | | | | | | | | | |  |
|  | **OR** | **95% CI** | **p** |  | **OR** | **95% CI** | **p** |  | **OR** | **95% CI** | **p** |  | **OR** | **95% CI** | **p** |  |
| **CPM** |  |  |  |  |  |  |  |  |  |  |  |  |  |  |  |  |
| 14 years | 0.81 | (0.62,1.06) | 0.13 |  | 0.78 | (0.59,1.03) | 0.08 |  | 0.86 | (0.63,1.17) | 0.33 |  | 0.81 | (0.59,1.11) | 0.19 |  |
| **MVPA** |  |  |  |  |  |  |  |  |  |  |  |  |  |  |  |  |
| 14 years | 0.64 | (0.38,1.06) | 0.08 |  | 0.61 | (0.36,1.05) | 0.08 |  | 0.71 | (0.39,1.27) | 0.25 |  | 0.66 | (0.37,1.18) | 0.17 |  |
| **USS Liver Stiffness** | | | | | | | | | | | | | | | |  |
|  | **% Change** | **95% CI** | **p** |  | **% Change** | **95% CI** | **p** |  | **% Change** | **95% CI** | **p** |  | **% Change** | **95% CI** | **p** |  |
| **CPM** |  |  |  |  |  |  |  |  |  |  |  |  |  |  |  |  |
| 14 years | 0% | (-1%,0%) | 0.56 |  | 0% | (-1%,0%) | 0.34 |  | 0% | (-1%,0%) | 0.57 |  | 0% | (-1%,0%) | 0.38 |  |
| **MVPA** |  |  |  |  |  |  |  |  |  |  |  |  |  |  |  |  |
| 14 years | 0% | (-1%,1%) | 0.48 |  | -1% | (-2%,1%) | 0.32 |  | 0% | (-1%,1%) | 0.51 |  | -1% | (-2%,1%) | 0.33 |  |
| Coefficients are per increase of 100 CPM or per 15 minute increase in MVPA. Model 1 is unadjusted. Model 2 adjusts for mother’s age at delivery, parity, sex, ethnicity, mother’s education, head of household social class, mother’s BMI, energy intake at age 11 years, pubertal status at age 11, age at physical activity assessment, length of time accelerometer was worn (in minutes) and age at the time of liver assessment. Model 3 is the same as model 2 but additionally adjusts for fat mass, height and height^2^ at the time physical activity was assessed. Model 4 is the same as model 2 but additionally adjusts for fat mass, height and height^2^ at the time of liver outcome assessment. | | | | | | | | | | | | | | | | |

**Table L: Associations of CPM and MVPA at age 14 years with blood-based liver outcomes at mean age 17.8 years in the imputed data restricted to participants with physical activity data at age 14 (n=2000)**

|  | **Model 1** | | |  | **Model 2** | | |  | **Model 3** | | |  | **Model 4** | | |  |
| --- | --- | --- | --- | --- | --- | --- | --- | --- | --- | --- | --- | --- | --- | --- | --- | --- |
| **ALT** | | | | | | | | | | | | | | | | |
|  | **% Change** | **95% CI** | **p** |  | **% Change** | **95% CI** | **p** |  | **% Change** | **95% CI** | **p** |  | **% Change** | **95% CI** | **p** |  |
| **CPM** |  |  |  |  |  |  |  |  |  |  |  |  |  |  |  |  |
| 14 years | 1% | (0%,2%) | 0.03 |  | 0% | (-1%,1%) | 0.68 |  | 0% | (-1%,1%) | 0.58 |  | 0% | (-1%,1%) | 0.45 |  |
| **MVPA** |  |  |  |  |  |  |  |  |  |  |  |  |  |  |  |  |
| 14 years | 1% | (-1%,3%) | 0.24 |  | -1% | (-3%,1%) | 0.23 |  | 0% | (-2%,1%) | 0.65 |  | 0% | (-2%,2%) | 0.91 |  |
| **AST** | | | | | | | | | | | | | | | | |
|  | **% Change** | **95% CI** | **p** |  | **% Change** | **95% CI** | **p** |  | **% Change** | **95% CI** | **p** |  | **% Change** | **95% CI** | **p** |  |
| **CPM** |  |  |  |  |  |  |  |  |  |  |  |  |  |  |  |  |
| 14 years | 2% | (1%,2%) | <0.01 |  | 1% | (0%,1%) | 0.08 |  | 1% | (0%,1%) | 0.12 |  | 1% | (0%,1%) | 0.04 |  |
| **MVPA** |  |  |  |  |  |  |  |  |  |  |  |  |  |  |  |  |
| 14 years | 3% | (1%,4%) | <0.01 |  | 1% | (0%,2%) | 0.10 |  | 1% | (0%,2%) | 0.12 |  | 1% | (0%,2%) | 0.05 |  |
| **GGT** | | | | | | | | | | | | | | | | |
|  | **% Change** | **95% CI** | **p** |  | **% Change** | **95% CI** | **p** |  | **% Change** | **95% CI** | **p** |  | **% Change** | **95% CI** | **p** |  |
| **CPM** |  |  |  |  |  |  |  |  |  |  |  |  |  |  |  |  |
| 14 years | 1% | (0%,2%) | 0.03 |  | -1% | (-2%,0%) | 0.09 |  | 0% | (-1%,1%) | 0.65 |  | 0% | (-1%,1%) | 0.70 |  |
| **MVPA** |  |  |  |  |  |  |  |  |  |  |  |  |  |  |  |  |
| 14 years | -1% | (-2%,1%) | 0.31 |  | -1% | (-2%,1%) | 0.29 |  | 0% | (-1%,1%) | 0.92 |  | 0% | (-1%,2%) | 0.84 |  |
| Coefficients are per increase of 100 CPM or per 15 minute increase in MVPA. Model 1 is unadjusted. Model 2 adjusts for mother’s age at delivery, parity, sex, ethnicity, mother’s education, head of household social class, mother’s BMI, energy intake at age 11 years, pubertal status at age 11, age at physical activity assessment, length of time accelerometer was worn (in minutes) and age at the time of liver assessment. Model 3 is the same as model 2 but additionally adjusts for fat mass, height and height^2^ at the time physical activity was assessed. Model 4 is the same as model 2 but additionally adjusts for fat mass, height and height^2^ at the time of liver outcome assessment. | | | | | | | | | | | | | | | | |

**Table M: Associations of missing physical activity data at age 14 with CPM and MVPA at age 12**

|  | **Missing physical activity at age 14** | | | | | | |
| --- | --- | --- | --- | --- | --- | --- | --- |
|  | **USS dataset (n=162)** | | |  | **Biomarker dataset (n=533)** | | |
|  | **OR** | **95% CI** | **p** |  | **OR** | **95% CI** | **p** |
| **CPM at age 12** | 0.93^a^ | 0.39 to 2.21 | 0.87 |  | 1.12^c^ | 0.70 to 1.81 | 0.63 |
|  |  |  |  |  |  |  |  |
| **MVPA at age 12** | 0.40^b^ | 0.06 to 2.64 | 0.34 |  | 0.46^d^ | 0.19 to 1.81 | 0.10 |
| ^a^ Adjusted for MVPA at age 12; total minutes wearing the accelerometer at ages 12 and 14; USS liver fat, stiffness and volume, tanner pubic hair stage, fat mass, height, height squared, AUDIT scores and HOMA-IR at outcome assessment (mean age 17.8 years); age, weight and height at 12, 14 and 15 year follow-up clinics; energy intake at 11; maternal age, parity, maternal BMI, maternal education, head of household social class, sex, ethnicity and AUDIT scores at age 16 ^b^ Adjusted for CPM at age 12; total minutes wearing the accelerometer at ages 12 and 14; USS liver fat, stiffness and volume, tanner pubic hair stage, fat mass, height, height squared, AUDIT scores and HOMA-IR at outcome assessment (mean age 17.8 years); age, weight and height at 12, 14 and 15 year follow-up clinics; energy intake at 11; maternal age, parity, maternal BMI, maternal education, head of household social class, sex, ethnicity and AUDIT scores at age 16 ^c^ Adjusted for MVPA at age 12; total minutes wearing the accelerometer at ages 12 and 14; ALT, AST, GGT, haptoglobin, tanner pubic hair stage, fat mass, height, height squared, AUDIT scores and HOMA-IR at outcome assessment (mean age 17.8 years); age, weight and height at 12, 14 and 15 year follow-up clinics; energy intake at 11; maternal age, parity, maternal BMI, maternal education, head of household social class, sex, ethnicity and AUDIT scores at age 16 ^d^ Adjusted for CPM at age 12; total minutes wearing the accelerometer at ages 12 and 14; ALT, AST, GGT, haptoglobin, tanner pubic hair stage, fat mass, height, height squared, AUDIT scores and HOMA-IR at outcome assessment (mean age 17.8 years); age, weight and height at 12, 14 and 15 year follow-up clinics; energy intake at 11; maternal age, parity, maternal BMI, maternal education, head of household social class, sex, ethnicity and AUDIT scores at age 16 | | | | | | | |

**Table N: Associations of CPM and MVPA at ages 12 and 14 years with USS liver outcomes at mean age 17.8 years in the imputed data, with additional adjustment for AUDIT scores (n=1292)**

|  | **Model 1** | | |  | **Model 2** | | |  |  | **Model 3** |  | **Model 4** | | |  |
| --- | --- | --- | --- | --- | --- | --- | --- | --- | --- | --- | --- | --- | --- | --- | --- |
| **USS Liver Fat** | | | | | | | | | | | | | | | |
|  | **OR** | **95% CI** | **p** |  | **OR** | **95% CI** | **p** |  | **OR** | **95% CI** | **p** | **OR** | **95% CI** | **p** |  |
| **CPM** |  |  |  |  |  |  |  |  |  |  |  |  |  |  |  |
| 12 years | 0.76 | (0.58,0.98) | 0.03 |  | 0.71 | (0.53,0.95) | 0.02 |  | 0.83 | (0.60,1.13) | 0.24 | 0.76 | (0.56,1.04) | 0.09 |  |
| 14 years | 0.79 | (0.60,1.04) | 0.10 |  | 0.76 | (0.57,1.01) | 0.06 |  | 0.83 | (0.61,1.15) | 0.26 | 0.81 | (0.60,1.10) | 0.18 |  |
| **MVPA** |  |  |  |  |  |  |  |  |  |  |  |  |  |  |  |
| 12 years | 0.55 | (0.32,0.94) | 0.03 |  | 0.47 | (0.26,0.85) | 0.01 |  | 0.66 | (0.35,1.25) | 0.21 | 0.60 | (0.32,1.12) | 0.11 |  |
| 14 years | 0.63 | (0.39,1.02) | 0.06 |  | 0.63 | (0.38,1.03) | 0.06 |  | 0.72 | (0.41,1.26) | 0.25 | 0.69 | (0.41,1.18) | 0.18 |  |
| **USS Liver Stiffness** | | | | | | | | | | | | | | | |
|  | **% Change** | **95% CI** | **p** |  | **% Change** | **95% CI** | **p** |  | **% Change** | **95% CI** | **p** | **% Change** | **95% CI** | **p** |  |
| **CPM** |  |  |  |  |  |  |  |  |  |  |  |  |  |  |  |
| 12 years | 0% | (-1%,1%) | 0.87 |  | 0% | (-1%,1%) | 0.98 |  | 0% | (0%,1%) | 0.37 | 0% | (-1%,1%) | 0.69 |  |
| 14 years | 0% | (-1%,0%) | 0.18 |  | 0% | (-1%,0%) | 0.19 |  | 0% | (-1%,0%) | 0.41 | 0% | (-1%,0%) | 0.25 |  |
| **MVPA** |  |  |  |  |  |  |  |  |  |  |  |  |  |  |  |
| 12 years | 0% | (-1%,1%) | 0.65 |  | 0% | (-1%,1%) | 0.81 |  | 1% | (-1%,2%) | 0.39 | 0% | (-1%,1%) | 0.75 |  |
| 14 years | -1% | (-2%,0%) | 0.15 |  | -1% | (-2%,0%) | 0.21 |  | 0% | (-1%,1%) | 0.39 | -1% | (-2%,0%) | 0.24 |  |
| Coefficients are per increase of 100 CPM or per 15 minute increase in MVPA. Model 1 is unadjusted. Model 2 adjusts for mother’s age at delivery, parity, sex, ethnicity, mother’s education, head of household social class, mother’s BMI, energy intake at age 11 years, pubertal status at age 11, age at physical activity assessment, length of time accelerometer was worn (in minutes), AUDIT scores at 16 and 17-18y follow-up assessments and age at the time of liver assessment. Model 3 is the same as model 2 but additionally adjusts for fat mass, height and height^2^ at the time physical activity was assessed. Model 4 is the same as model 2 but additionally adjusts for fat mass, height and height^2^ at the time of liver outcome assessment. | | | | | | | | | | | | | | | |

**Table O: Associations of CPM and MVPA at ages 12 and 14 years with blood-based liver outcomes at mean age 17.8 years in the imputed data, with additional adjustment for AUDIT scores (n=2612)**

|  | **Model 1** | | |  | **Model 2** | | |  | **Model 3** |  |  | **Model 4** | | |  |
| --- | --- | --- | --- | --- | --- | --- | --- | --- | --- | --- | --- | --- | --- | --- | --- |
| **ALT** | | | | | | | | | | | | | | | |
|  | **% Change** | **95% CI** | **p** |  | **% Change** | **95% CI** | **p** | **% Change** | **95% CI** | **p** |  | **% Change** | **95% CI** | **p** |  |
| **CPM** |  |  |  |  |  |  |  |  |  |  |  |  |  |  |  |
| 12 years | 1% | (0%,2%) | 0.05 |  | 0% | (-1%,1%) | 0.62 | 1% | (-1%,2%) | 0.33 |  | 1% | (0%,2%) | 0.27 |  |
| 14 years | 1% | (0%,2%) | 0.03 |  | 0% | (-1%,1%) | 0.56 | 0% | (-1%,1%) | 0.77 |  | 0% | (-1%,1%) | 0.60 |  |
| **MVPA** |  |  |  |  |  |  |  |  |  |  |  |  |  |  |  |
| 12 years | 1% | (-1%,3%) | 0.20 |  | -1% | (-3%,1%) | 0.18 | 0% | (-2%,2%) | 0.88 |  | 1% | (-1%,2%) | 0.56 |  |
| 14 years | 1% | (-1%,3%) | 0.20 |  | -1% | (-3%,1%) | 0.22 | 0% | (-2%,1%) | 0.59 |  | 0% | (-2%,2%) | 0.84 |  |
| **AST** | | | | | | | | | | | | | | | |
|  | **% Change** | **95% CI** | **p** |  | **% Change** | **95% CI** | **p** | **% Change** | **95% CI** | **p** |  | **% Change** | **95% CI** | **p** |  |
| **CPM** |  |  |  |  |  |  |  |  |  |  |  |  |  |  |  |
| 12 years | 2% | (1%,2%) | <0.01 |  | 2% | (1%,2%) | <0.01 | 1% | (0%,1%) | 0.05 |  | 1% | (0%,1%) | 0.07 |  |
| 14 years | 2% | (1%,2%) | <0.01 |  | 2% | (1%,2%) | <0.01 | 1% | (0%,1%) | 0.17 |  | 0% | (0%,1%) | 0.21 |  |
| **MVPA** |  |  |  |  |  |  |  |  |  |  |  |  |  |  |  |
| 12 years | 3% | (2%,4%) | <0.01 |  | 1% | (0%,2%) | 0.10 | 1% | (0%,2%) | 0.11 |  | 1% | (0%,3%) | 0.03 |  |
| 14 years | 3% | (1%,4%) | <0.01 |  | 1% | (0%,2%) | 0.21 | 1% | (0%,2%) | 0.22 |  | 1% | (0%,2%) | 0.13 |  |
| **GGT** | | | | | | | | | | | | | | | |
|  | **% Change** | **95% CI** | **p** |  | **% Change** | **95% CI** | **p** | **% Change** | **95% CI** | **p** |  | **% Change** | **95% CI** | **p** |  |
| **CPM** |  |  |  |  |  |  |  |  |  |  |  |  |  |  |  |
| 12 years | 0% | (-1%,1%) | 0.46 |  | -1% | (-2%,-1%) | <0.01 | -1% | (-2%,0%) | 0.10 |  | -1% | (-1%,0%) | 0.11 |  |
| 14 years | 1% | (0%,2%) | 0.02 |  | -1% | (-2%,0%) | 0.10 | 0% | (-1%,1%) | 0.51 |  | 0% | (-1%,1%) | 0.60 |  |
| **MVPA** |  |  |  |  |  |  |  |  |  |  |  |  |  |  |  |
| 12 years | 1% | (-1%,2%) | 0.42 |  | -3% | (-4%,-2%) | <0.01 | -2% | (-3%,0%) | 0.02 |  | -1% | (-3%,0%) | 0.06 |  |
| 14 years | 2% | (0%,3%) | 0.02 |  | -1% | (-2%,0%) | 0.16 | 0% | (-2%,1%) | 0.55 |  | 0% | (-2%,1%) | 0.80 |  |
| Coefficients are per increase of 100 CPM or per 15 minute increase in MVPA. Model 1 is unadjusted. Model 2 adjusts for mother’s age at delivery, parity, sex, ethnicity, mother’s education, head of household social class, mother’s BMI, energy intake at age 11 years, pubertal status at age 11, age at physical activity assessment, length of time accelerometer was worn (in minutes) AUDIT scores at 16 and 17-18y follow-up assessments and age at the time of liver assessment. Model 3 is the same as model 2 but additionally adjusts for fat mass, height and height^2^ at the time physical activity was assessed. Model 4 is the same as model 2 but additionally adjusts for fat mass, height and height^2^ at the time of liver outcome assessment. | | | | | | | | | | | | | | | |

**Reference List**

1. Anderson EL, Tilling K, Fraser A et al. Estimating trajectories of energy intake through childhood and adolescence using linear-spline multilevel models. Epidemiology 2013;24:507-15.

2. Marshall WA, Tanner JM. Variations in the Pattern of Pubertal Changes in Boys. Archives of Disease in Childhood 1970;45:13-23.

3. Marshall WA, Tanner JM. Variations in pattern of pubertal changes in girls. Arch Dis Child 1969;44:291-303.

4. Royston P. Multiple imputation of missing values. Stata J 2004;4:227-41.
